# Teriparatide in Two Patients With Mucopolysaccharidosis Type IVB

**DOI:** 10.1002/jmd2.70088

**Published:** 2026-04-13

**Authors:** Mark Wijnen, Evert F. S. van Velsen, J. Gert‐Jan Milhous, Esmee Oussoren, Bram C. J. van der Eerden, Margreet A. E. M. Wagenmakers

**Affiliations:** ^1^ Department of Internal Medicine, Center for Lysosomal and Metabolic Diseases Erasmus Medical Center Rotterdam the Netherlands; ^2^ Department of Internal Medicine, Section Endocrinology, Erasmus MC Bone Center Erasmus Medical Center Rotterdam the Netherlands; ^3^ Department of Cardiology Admiraal de Ruyter Hospital in Goes, Vlissingen, and Zierikzee the Netherlands; ^4^ Department of Pediatrics, Center for Lysosomal and Metabolic Diseases Erasmus Medical Center Rotterdam the Netherlands; ^5^ Laboratory for Calcium and Bone Metabolism, Department of Internal Medicine Erasmus Medical Center Rotterdam the Netherlands

**Keywords:** aortic stenosis, cardiovascular disease, low bone mineral density, mucopolysaccharidosis, osteoporosis, teriparatide

## Abstract

Mucopolysaccharidosis Type IV is a multisystem lysosomal storage disease characterized by severe skeletal dysplasia resulting from impaired degradation of the glycosaminoglycans keratan sulfate and chondroitin‐6‐sulfate. The condition is classified into Types A and B based on the underlying enzyme deficiency. Low bone mineral density (BMD) is a feature of the skeletal phenotype, contributing to increased fracture risk. Extraskeletal manifestations include, among others, cardiovascular disease due to valvular stenosis and regurgitation, myocardial remodeling, coronary artery disease, and vascular stiffness, all associated with glycosaminoglycan accumulation. We report two adult patients with mucopolysaccharidosis Type IVB treated with the osteoanabolic agent teriparatide for an apparently low BMD. The first patient presented with a non‐healing femoral fracture requiring BMD improvement prior to surgical fixation. This patient was treated with teriparatide for 2 years, which resulted in significant BMD gain, enabling successful surgical fixation. The second patient received teriparatide for only 6 months and showed stable BMD. Notably, both patients developed serious cardiac problems during teriparatide treatment: the first patient experienced rapidly progressive aortic stenosis, while the second patient developed dyspnea and polyuria due to worsening dynamic left ventricular outflow tract obstruction, which had previously been asymptomatic. Both patients required invasive cardiac interventions, which carry high risk in mucopolysaccharidosis due to unique anatomical and anesthetic challenges. The temporal association between teriparatide treatment and the onset of cardiac problems in both patients is noteworthy and raises the possibility of a treatment‐related effect. Therefore, we recommend cautious use of teriparatide in patients with mucopolysaccharidosis Type IV.

## Key Points

- This is the first reported use of teriparatide in patients with mucopolysaccharidosis Type IVB.
- Teriparatide treatment was able to improve bone mineral density.
- Both patients developed serious cardiac problems during teriparatide treatment, requiring invasive cardiac interventions, and underscoring the need for caution when considering teriparatide in mucopolysaccharidosis.

## 1 Introduction

Mucopolysaccharidosis Type IV is a multisystem lysosomal storage disease caused by autosomal recessive deficiency of glycosaminoglycan‐degrading enzymes: *N*‐acetylgalactosamine‐6‐sulfatase in mucopolysaccharidosis Type IVA and *β*‐galactosidase in mucopolysaccharidosis Type IVB. The resulting accumulation of keratan sulfate (in both types) and chondroitin‐6‐sulfate (in Type IVA only) leads to the characteristic phenotype [1].

Skeletal dysplasia is the hallmark of mucopolysaccharidosis Type IV and is characterized by spondyloepiphyseal abnormalities, including vertebral malformations with odontoid hypoplasia, paddle‐shaped ribs, and dysplasia of the metaphyses and epiphyses. Collectively, these abnormalities are referred to as dysostosis multiplex and result in short‐trunk dwarfism [2]. Beyond these overt skeletal changes, low bone mineral density (BMD) is common and contributes to increased fracture risk [3]. Extraskeletal manifestations of mucopolysaccharidosis Type IV include corneal clouding, sensorineural hearing loss, dental anomalies, upper airway obstruction, hepatomegaly, and cardiovascular disease [1]. Cardiovascular disease typically includes valvular stenosis and regurgitation (primarily aortic and mitral), myocardial remodeling, coronary artery disease, and vascular stiffness, all associated with glycosaminoglycan accumulation [4].

Although no curative treatment for mucopolysaccharidosis Type IV exists, enzyme replacement therapy with recombinant human *N*‐acetylgalactosamine‐6‐sulfatase (elosulfase alfa) has been available for mucopolysaccharidosis Type IVA since 2014 [5]. Its impact on skeletal manifestations appears to be modest. In a retrospective cohort of 17 patients treated for a median of 7.4 years, all 12 individuals who reached final adult height while receiving enzyme replacement therapy exhibited severely reduced stature, remaining below the third percentile [6].

Bone pathology in mucopolysaccharidosis Type IV results from the accumulation of keratan sulfate, and in type IVA, chondroitin‐6‐sulfate. Both glycosaminoglycans are primarily synthesized in cartilage, and their storage within cartilage and the surrounding extracellular matrix disrupts normal chondrogenesis and endochondral ossification [7]. These disruptions, combined with reduced mobility due to physical impairments, contribute to low BMD [3].

Therapeutic options for low BMD include antiresorptives (bisphosphonates and denosumab), which inhibit osteoclast‐mediated bone resorption, and osteoanabolic agents (teriparatide, abaloparatide, and romosozumab), which stimulate osteoblast activity to promote bone formation. Romosozumab exerts dual action by also inhibiting osteoclast function [8]. Osteoanabolic agents generally demonstrate superior efficacy in increasing BMD and reducing fracture risk compared with antiresorptives [9].

We report two adult patients with mucopolysaccharidosis Type IVB who were treated with teriparatide for low BMD. Although teriparatide was able to improve BMD, both patients developed serious cardiac problems, requiring invasive cardiac interventions. Treatment decisions regarding bone‐strengthening therapy in both patients were based solely on BMD values unadjusted for height (height‐adjusted BMD values were not considered at treatment initiation nor during the course of therapy).

## 2 Case Series

### 2.1 Patient 1

The first patient is a 60‐year‐old male diagnosed with mucopolysaccharidosis Type IVB at 3 years of age (*β*‐galactosidase activity in leukocytes: 0.06 nmol/mg protein/min [reference range: 2.46 ± 0.85]). His genotype showed homozygosity for the *GLB1* variant c.817_818delinsCT, p.(Trp273Leu). His medical history included hypertension and left retinal detachment.

At 57 years of age, he was referred to our inherited metabolic disease outpatient clinic by his primary care physician because of urinary and fecal incontinence. Neurological evaluation revealed spinal cord compression secondary to dysostosis multiplex and vertebral disc bulging as the underlying cause. Clean intermittent catheterization was initiated for urinary incontinence, and supportive measures were provided for fecal incontinence. The patient also reported progressive pain and swelling of the right leg, which had developed spontaneously 1 year prior and ultimately resulted in loss of ambulation.

Ultrasound of the right leg revealed a subcutaneous bony structure near the distal femur, prompting radiographic evaluation. X‐ray imaging confirmed a non‐healing femoral fracture. The patient was referred to an orthopedic surgeon, and alendronate 70 mg once weekly was initiated. The patient had no history of prior fractures, and investigations for underlying causes of low BMD showed no abnormalities (Table 1). Meanwhile, a comprehensive multisystem evaluation was performed to assess (pre)symptomatic manifestations of mucopolysaccharidosis Type IVB. Transthoracic echocardiography revealed asymptomatic, non‐severe aortic stenosis (mean pressure gradient: 30 mmHg; peak transvalvular velocity: 3.4 m/s; aortic valve area: 1.0 cm^2^). Dual‐energy X‐ray absorptiometry (DEXA) demonstrated a reduced BMD (lumbar spine: 0.718 g/cm^2^ [*T*‐score: −4.0 SD; *Z*‐score: −3.1 SD; height‐adjusted *Z*‐score: −0.7 SD]; left femoral neck: 0.211 g/cm^2^ [*T*‐score: −6.0 SD; *Z*‐score: −5.8 SD; height‐adjusted *Z*‐score: −4.4 SD]). Given the markedly low BMD of the left femoral neck, surgical fracture fixation was deemed too high‐risk due to potential osteosynthesis failure.

**TABLE 1:** Results of laboratory investigations evaluating potential underlying causes of low bone mineral density prior to teriparatide treatment.

	Patient 1 (M)	Patient 2 (F)	Reference values
Cystatin C‐based eGFR (mL/min)	53	73	> 90
Calcium (mmol/L)	2.47	2.42	2.20–2.65
Albumin (g/L)	39	42	35–50
Phosphate (mmol/L)	0.95	1.19	0.80–1.40
Alkaline phosphatase (U/L)	155	125	< 115 (M) < 98 (F)
γ‐glutamyltransferase (U/L)	18	23	< 55 (M) < 38 (F)
25‐hydroxy vitamin D (nmol/L)	66	57	50–120
PTH (pmol/L)	8.6	5.0	0.68–4.40

Abbreviations: eGFR = estimated glomerular filtration rate, F = female, M = male, PTH = parathyroid hormone.

Because the anticipated BMD improvement with alendronate was considered insufficient, therapy was switched to teriparatide (20 μg subcutaneously once daily) after 3 months and continued for 2 years. After 2 years of teriparatide treatment, BMD had improved significantly (lumbar spine: +24.1% [0.891 g/cm^2^; *T*‐score: −2.6 SD; *Z*‐score: −1.6 SD; height‐adjusted *Z*‐score: +0.8 SD], left femoral neck: +52.6% [0.322 g/cm^2^; *T*‐score: −5.2 SD; *Z*‐score: −4.9 SD; height‐adjusted *Z*‐score: −3.5 SD]) (Table 2). Subsequently, the patient underwent successful surgical fracture fixation. BMD improvement was consolidated with a zoledronic acid infusion (5 mg).

**TABLE 2:** Longitudinal changes in bone mineral density during teriparatide.

	Before teriparatide^a^	One year after teriparatide initiation	Two years after teriparatide initiation
Patient 1			
Teriparatide duration (month)	24		
Height (cm)	158		
BMD			
Lumbar spine			
% versus baseline		+12.8^b^	+24.1^b^
g/cm^2^	0.718	0.810	0.891
*T*‐score (SD)	−4.0	−3.6	−2.6
*Z*‐score (SD)	−3.1	−2.3	−1.6
HAZ *Z*‐score (SD)	−0.7	+0.1	+0.8
Left femoral neck			
% versus baseline		+146^c^	+52.6^b^
g/cm^2^	0.211	0.520	0.322
*T*‐score (SD)	−6.0	−4.3	−5.2
*Z*‐score (SD)	−5.8	−2.8	−4.9
HAZ *Z*‐score (SD)	−4.4	−1.4	−3.5
Patient 2			
Teriparatide duration (mo.)	6		
Height (cm)	132		
BMD			
Lumbar spine			
% versus baseline		+1.8	
g/cm^2^	0.741	0.755	
*T*‐score (SD)	−3.8	−3.7	
*Z*‐score (SD)	−2.2	−2.0	
HAZ *Z*‐score (SD)	+1.8	+2.0	
Distal radius of non‐dominant forearm			
% versus baseline		0	
g/cm^2^	0.598	0.598	
*T*‐score (SD)	−3.3	−3.3	
*Z*‐score (SD)	−3.0	−2.8	
HAZ *Z*‐score (SD)	−0.1	+0.1	

*Note:* All scans were performed on the same Lunar Prodigy Advance machine. Bone mineral density *Z*‐scores adjusted for height‐for‐age *Z*‐score were calculated according to the method described by Zemel et al. [10] CHeight‐for‐age *Z*‐scores were calculated using Dutch reference data from the Netherlands Organization for Applied Scientific Research (TNO) [11].

Abbreviations: BMD = bone mineral density; HAZ = height‐for‐age Z‐score; mo. = months; SD = standard deviation.

^a^ Dual energy X‐ray absorptiometry (DEXA) was performed 6 months prior to teriparatide initiation in Patient 1 and 1 month prior to teriparatide initiation in Patient 2.

^b^ Significant difference compared with baseline.

^c^ Value overestimated due to different patient positioning during DEXA.

**FIGURE 1:**
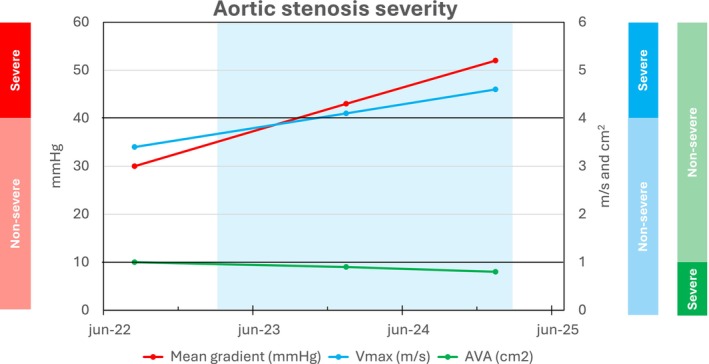
Progression of aortic stenosis during teriparatide treatment in Patient 1. The shaded area indicates the period of teriparatide treatment. Aortic stenosis severity is classified according to the 2025 ESC/EACTS Guidelines for the Management of Valvular Heart Disease by Praz et al. [12].

One year after teriparatide initiation, surveillance echocardiography revealed progression to severe aortic stenosis (mean pressure gradient: 43 mmHg; peak transvalvular velocity: 4.1 m/s; aortic valve area: 0.9 cm^2^) (Figure 1). As the patient remained asymptomatic, follow‐up echocardiography was scheduled for 6 months later. Due to patient delay, follow‐up echocardiography was performed after 1 year, coinciding with completion of 2 years of teriparatide treatment. Echocardiography revealed further progression of severe aortic stenosis (mean pressure gradient: 52 mmHg; peak transvalvular velocity: 4.6 m/s; aortic valve area: 0.8 cm^2^). Although the patient remained asymptomatic, the rapid progression prompted valve replacement.

A transcatheter aortic valve implantation work‐up was performed, including coronary angiography, which showed no significant coronary artery stenosis, and cardiovascular computed tomography, which demonstrated an aortic valve calcium score of 7550 Agatston units (> 2000 indicates severe calcific aortic stenosis [12]) and a tortuous descending thoracic aorta. Due to the tortuosity, percutaneous valve replacement was deemed unsuitable. The patient was referred to a cardiothoracic surgeon and subsequently underwent an uneventful minimally invasive surgical aortic valve replacement.

### 2.2 Patient 2

The second patient is a 55‐year‐old female diagnosed with mucopolysaccharidosis Type IVB at 4 years of age (*β*‐galactosidase activity in leukocytes: 5 nmol/h/mg protein [reference range: 50–326]). Her *GLB1* genotype showed compound heterozygosity for the variants c.245+1G>T, p.(?) and c.817_818delinsCT, p.(Trp273Leu). Her medical history included bilateral total hip replacement 11 years prior for severe osteoarthritis, resulting in wheelchair dependency. Additionally, 2 years prior, she had been diagnosed with asymptomatic, non‐severe aortic stenosis (mean pressure gradient: 23 mmHg; peak transvalvular velocity: 3.4 m/s; aortic valve area: 1.1 cm^2^).

At 46 years of age, she was referred to our inherited metabolic disease outpatient clinic by her primary care practitioner for multisystem evaluation of (pre)symptomatic features of mucopolysaccharidosis Type IVB. At the time of referral, she reported no complaints. Transthoracic echocardiography revealed slight progression of her non‐severe aortic stenosis (mean pressure gradient: 34 mmHg; peak transvalvular velocity: 3.7 m/s; aortic valve area: 1.3 cm^2^) and a newly identified non‐severe mitral stenosis (maximal gradient: 18 mmHg; mean gradient: 8 mmHg; heart rate: 69 bpm). Annual surveillance echocardiography was scheduled and revealed gradual development of concentric left ventricular hypertrophy with left ventricular outflow tract obstruction over subsequent years, complicating reliable assessment of aortic stenosis severity. Non‐severe mitral stenosis remained stable during 8 years of follow‐up (maximal gradient: 22 mmHg; mean gradient: 9 mmHg; heart rate: 79 bpm at last echocardiography).

DEXA at the initial referral demonstrated an apparently reduced BMD (lumbar spine: 0.697 g/cm^2^ [*T*‐score: −4.2 SD; *Z*‐score: −2.0 SD; height‐adjusted *Z*‐score: +2.0 SD]). No underlying causes of low BMD were identified on laboratory investigation (Table 1). Although the patient had not sustained any fractures, alendronate 70 mg once weekly was initiated. After 5 years of alendronate, follow‐up DEXA demonstrated no BMD improvement (lumbar spine: 0.741 g/cm^2^ [*T*‐score: −3.8 SD; *Z*‐score: −2.2 SD; height‐adjusted *Z*‐score: +1.8 SD], distal radius of the non‐dominant forearm: 0.598 g/cm^2^ [*T*‐score: −3.3 SD; *Z*‐score: −3.0 SD; height‐adjusted *Z*‐score: −0.1 SD]). Given the modest response, alendronate was switched to teriparatide. Six months after initiating teriparatide, the patient reported joint pain, dyspnea, and polyuria. Consequently, teriparatide was discontinued and alendronate restarted. Follow‐up DEXA 6 months after discontinuing teriparatide revealed stable BMD (lumbar spine: +1.8% [0.755 g/cm^2^; *T*‐score: −3.7 SD; *Z*‐score: −2.0 SD; height‐adjusted *Z*‐score: +2.0 SD], distal radius of the non‐dominant forearm: 0% [0.598 g/cm^2^; *T*‐score: −3.3 SD; *Z*‐score: −2.8 SD; height‐adjusted *Z*‐score: +0.1 SD]) (Table 2).

One month before teriparatide discontinuation (5 months after teriparatide initiation), a surveillance echocardiogram revealed an unexpected rise in the aortic valve mean pressure gradient (48 vs. 36 mmHg 6 months prior) and peak transvalvular velocity (4.5 vs. 3.9 m/s 6 months prior). The cardiologist attributed these changes to worsening dynamic left ventricular outflow tract obstruction, contributing to the patient's symptoms of dyspnea and polyuria. Furosemide 20 mg once daily was tried but did not relieve the symptoms.

A cardiac septal reduction procedure was deemed necessary. A percutaneous transluminal septal myocardial ablation was attempted but was unsuccessful due to small septal perforating arteries. Subsequently, an uneventful septal scoring along the midline endocardium procedure was performed, successfully relieving obstruction. This resulted in resolution of the symptoms, allowing discontinuation of furosemide. A follow‐up echocardiogram is scheduled 6 months post‐procedure and has yet to be performed.

## 3 Discussion

This is the first report of teriparatide treatment in mucopolysaccharidosis type IVB. Two years of teriparatide treatment resulted in significant BMD improvement in Patient 1, enabling successful surgical fracture fixation. In Patient 2, BMD remained stable after 6 months of treatment. Importantly, both patients developed serious cardiac problems during teriparatide treatment, requiring invasive cardiac interventions.

Teriparatide is a recombinant human parathyroid hormone analog, comprising the bioactive 34‐amino acid fragment of the full‐length hormone. It is administered as a daily subcutaneous injection (20 μg) for up to 2 years [8]. Whereas continuous activation of the parathyroid hormone receptor, as occurs in primary hyperparathyroidism, promotes bone resorption, intermittent (pulsatile) stimulation—as achieved with teriparatide—enhances bone formation [13]. In Patient 1, BMD improved significantly after 2 years of treatment. In Patient 2, BMD remained stable, likely reflecting the short treatment duration of only 6 months. Because of altered bone morphology associated with mucopolysaccharidosis, direct comparison of BMD gains with those in the general population is not feasible [14]. Recognized adverse effects of teriparatide include extremity pain, nausea, headache, dizziness, and hypercalcemia [8]. Cardiovascular disease is not a known adverse effect, although two case reports have described rapid progression of pre‐existing aortic stenosis in patients without an inherited metabolic disease [15, 16].

Both of our patients with mucopolysaccharidosis developed cardiac problems during teriparatide treatment. Patient 1 experienced rapidly progressive aortic stenosis. Although no studies have specifically examined the natural history of aortic stenosis in mucopolysaccharidosis Type IV, one study evaluated long‐term progression of aortic stenosis in mucopolysaccharidosis Type II. In that cohort, among 12 patients not receiving enzyme replacement therapy, none developed severe aortic stenosis during a median follow‐up of 8.1 years (range, 2.6–17) [17].

In the pathophysiology of aortic stenosis, re‐differentiation of valvular interstitial cells into an osteoblast‐like phenotype that promotes valvular calcification is a key mechanism [18]. Teriparatide may potentiate this process by activating parathyroid hormone receptors on valvular endothelial cells. Receptor stimulation triggers p38 MAPK and ERK1/2 signaling pathways, leading to upregulation of BMP2 and RUNX2, which drive osteogenic transformation of valvular interstitial cells [19]. Specifically in patients with mucopolysaccharidosis, increased glycosaminoglycan accumulation may also contribute to progressive aortic stenosis. Activation of parathyroid hormone receptors has been shown to upregulate proteoglycan‐4 production [20], a molecule composed of a protein core with covalently attached glycosaminoglycan chains, which has been demonstrated to promote osteoblastic differentiation of valvular interstitial cells [21]. Both patients may have been more susceptible to these effects of parathyroid hormone receptor activation due to relatively high systemic teriparatide exposure, as no dose adjustments were made for altered pharmacokinetics associated with their small body size. Histopathological examination of Patient 1's native aortic valve could have provided valuable insights into the mechanisms underlying the rapidly progressive aortic stenosis; however, this was not possible because the excised valve was inadvertently not submitted for analysis. The treatment duration of only 6 months in Patient 2 may have been too short to trigger rapid progression of aortic stenosis in this patient.

Patient 2 developed progressive dyspnea and polyuria during teriparatide treatment, which appeared to be due to worsening dynamic left ventricular outflow tract obstruction that had previously been asymptomatic. Teriparatide may have precipitated these symptoms by increasing heart rate and lowering blood pressure [22, 23], effects mediated through positive chronotropic and vasodilatory actions of parathyroid hormone receptor activation [24]. Both tachycardia and vasodilation are well‐established precipitants of worsening dynamic left ventricular outflow tract obstruction [25]. Relatively high systemic teriparatide exposure, resulting from the absence of dose adjustment for small body size, might have increased Patient 2's susceptibility to these effects of parathyroid hormone receptor activation.

In both Patient 1 and Patient 2, BMD values unadjusted for height were used when deciding to initiate teriparatide treatment. However, in patients with short stature, such as those with mucopolysaccharidosis, BMD values may be underestimated if height adjustment is not applied [26]. Therefore, for both patients, we recalculated height‐for‐age‐adjusted BMD Z‐scores using the methodology described by Zemel et al. [10] and height‐for‐age *Z*‐score reference data from the Netherlands Organization for Applied Scientific Research [11]. These recalculations showed that Patient 2 had a BMD that was not lower than expected for her height. Combined with the absence of prior fractures, this suggests that teriparatide treatment may not have been necessary and could potentially have been avoided had height‐adjusted BMD been considered from the outset. This finding underscores the importance of incorporating height‐adjusted BMD values into the evaluation of patients with mucopolysaccharidosis.

The potential adverse cardiac effects of teriparatide are likely relevant for patients with other types of mucopolysaccharidosis as well, given shared underlying pathophysiological mechanisms. When osteoanabolic treatment is indicated, we advise against abaloparatide because it shares the same mechanism of action as teriparatide. Romosozumab, which acts through a different pathway, may represent an alternative [8].

In conclusion, we report two patients with mucopolysaccharidosis Type IVB who were treated with teriparatide. While teriparatide was able to improve BMD, both patients developed cardiac problems. The temporal association between teriparatide treatment and the onset of cardiac problems in both patients is noteworthy and raises the possibility of a treatment‐related effect. Accordingly, we advise cautious use of teriparatide in patients with mucopolysaccharidosis. However, given the presence of pre‐existing cardiac disease in both patients, the observed events may also reflect underlying pathology, and a causal relationship cannot be established. Future longitudinal studies are needed to further assess the safety of bone‐strengthening therapies in this population, to clarify the natural history of bone fragility and fracture risk, and to determine the most appropriate treatment strategy for low BMD in patients with mucopolysaccharidosis.

## Author Contributions

M. W. drafted the manuscript. E. F. S. v. V., J. G. J. M., E. O., B. C. J. v. d. E., and M. A. E. M. W. critically reviewed the manuscript. All authors approved the final version and agreed to its submission for publication.

## Funding

The authors have nothing to report.

## Ethics Statement

Both patients provided written informed consent for publication.

## Conflicts of Interest

E.F.S. van Velsen reports honoraria for lectures and consulting from Amgen, Theramex, UCB, and Kyowa Kirin. The other authors report no conflicts of interest.

## Data Availability

Data sharing not applicable to this article as no datasets were generated or analysed during the current study.
